# Retraction Note to: Correlates, comorbidities, and suicidal tendencies of problematic game use in a national wide sample of Korean adults

**DOI:** 10.1186/s13033-017-0162-2

**Published:** 2017-09-15

**Authors:** Subin Park, Hong Jin Jeon, Jung Woo Son, Haesoo Kim, Jin Pyo Hong

**Affiliations:** 1 Department of Research Planning, Mental Health Research Institute, National Center for Mental Health, Seoul, South Korea; 2 Department of Psychiatry, Samsung Medical Center, Sungkyunkwan University School of Medicine, 81 Irwon‑ro Gangnam‑gu, Seoul, 06351 Republic of Korea; 3 Department of Psychiatry, Chungbuk National University Hospital, Chungbuk National University School of Medicine, Cheongju, South Korea

## Retraction Note: Int J Ment Health Syst (2017) 11:35 DOI 10.1186/s13033-017-0143-5

The authors are retracting this article [1]. After publication they became aware that the criteria they had used for the diagnosis of problematic game use were not the DSM-5 Internet gaming disorder criteria as reported in the article but other similar criteria. As this error undermines the results and conclusions of their study they are retracting this article. All authors agree with this retraction.
